# Integrative analysis identifies Hspa5 as a key regulator of the ERS/UPR-immune axis in spinal cord injury

**DOI:** 10.3389/fgene.2026.1833119

**Published:** 2026-06-19

**Authors:** Guoyuan He, Aichun Yang, Hechun Xia

**Affiliations:** 1 School of Clinical Medicine, Ningxia Medical University, Yinchuan, China; 2 Ningxia Key Laboratory of Stem Cell and Regenerative Medicine, Institute of Medical Sciences, General Hospital of Ningxia Medical University, Yinchuan, China; 3 The First Clinical College, The First Affiliated Hospital, Hainan Medical University, Haikou, China; 4 Department of Neurosurgery, General Hospital of Ningxia Medical University, Yinchuan, China

**Keywords:** bioinformatics, endoplasmic reticulum stress, HSPA5, spinal cord injury, unfolded protein response

## Abstract

**Background:**

Endoplasmic reticulum stress (ERS) and the unfolded protein response (UPR) are critical in secondary spinal cord injury (SCI), but their systematic characterization and link to immune infiltration remain unclear. This study aimed to identify key ERS/UPR-related genes and their association with the immune microenvironment to find robust biomarkers for SCI.

**Methods:**

We analyzed bulk RNA-seq data (GSE151371) to identify differentially expressed genes (DEGs), followed by pathway enrichment analyses. Machine learning (SVM-RFE) and protein-protein interaction networks were used to screen for hub genes. The identified hub genes were validated using an independent cohort (GSE5296), single-cell sequencing data (GSE213240), and qRT-PCR in a murine SCI model.

**Results:**

We identified 17 ERS-related and 27 UPR-related DEGs, primarily enriched in neuron death pathways. SCI samples showed elevated ERS/UPR phenotype scores that positively correlated with myeloid cell infiltration. PPI analysis pinpointed *Dnajc3* and *Hspa5* as central hub genes. However, multi-level validation confirmed that while Hspa5 was consistently and significantly upregulated across all platforms, Dnajc3 did not show significant differential expression in single-cell sequencing and qRT-PCR validation, thereby limiting its credibility as a direct transcriptional biomarker. A diagnostic nomogram incorporating both genes achieved an AUC of 0.745.

**Conclusion:**

ERS/UPR activation is a critical component of the post-SCI transcriptomic response and is closely linked to immune remodeling. *Hspa5* emerges as a robust and dominant hub gene, representing a promising therapeutic target for SCI.

## 1 Introduction

Spinal cord injury (SCI) represents a profound neurological trauma that imposes long-term physical incapacity, significant psychological distress, and a heavy socioeconomic burden. The global scale of this issue has expanded markedly over the past 3 decades, with prevalence rates climbing from 236 to 1,298 per million people and an estimated 250,000 to 500,000 new injuries occurring each year (Khorasanizadeh et al., 2019). Lifetime costs per patient exceed 3 million dollars, and the annual economic burden reaches approximately 2.67 billion dollars in Canada (Katoh et al., 2019). The pathophysiology of SCI unfolds in two distinct temporal phases: an immediate primary injury and a subsequent, delayed secondary injury. The primary injury phase is initiated by the initial mechanical and biochemical insults to neural tissue, which directly provokes an inflammatory response and apoptotic cell death, marked by the rapid activation of resident microglia and infiltrating macrophages (Zhang et al., 2012; Zhang et al., 2019; Shi Z. et al., 2021). Secondary injury processes—encompassing inflammation, oxidative stress, ionic imbalance, and apoptosis-mediated cell death—collectively exacerbate tissue damage and lesion expansion, resulting in significant functional deficits (He et al., 2024; Ji et al., 2021; Liu et al., 2021). Endoplasmic reticulum stress (ERS) is another critical process closely associated with SCI. Triggers such as an excessive secretory load or the pathological buildup of malformed proteins can disrupt the homeostasis of the endoplasmic reticulum, leading to a state of stress (ERS). In response, cells initiate the unfolded protein response (UPR), an evolutionarily conserved adaptive pathway designed to counteract this dysfunction (Lin and Stone, 2020).

ERS has been identified as a key pathogenic factor in various neurological disorders, including SCI (Jingyu et al., 2018). The onset of ERS triggers the activation of three canonical ER-resident sensors—protein kinase RNA-like ER kinase (PERK), inositol-requiring enzyme 1 alpha (IRE1α), and activating transcription factor 6 (ATF6)—which collectively initiate a signaling cascade aimed at restoring proteostasis (Oakes and Papa, 2015). The PERK branch phosphorylates eukaryotic initiation factor 2α (eIF2α), transiently attenuating global translation (Harding et al., 1999; Liu et al., 2000); sustained PERK signaling upregulates C/EBP homologous protein (CHOP), promoting apoptosis (Mccullough et al., 2001; Marciniak et al., 2004). The IRE1α branch catalyzes unconventional splicing of X-box binding protein 1 (XBP1) mRNA to generate XBP1s, inducing ER chaperones and ER-associated degradation (ERAD) components (Yoshida et al., 2001; Calfon et al., 2002; Yamamoto et al., 2007). The ATF6 branch undergoes regulated intramembrane proteolysis in the Golgi, releasing a cytosolic fragment that upregulates protein folding and quality-control genes (Ye et al., 2000; Yamamoto et al., 2007). These three branches initially restore proteostasis; however, when ERS is severe or prolonged, the UPR shifts from a pro-survival to a pro-death program. Central to this switch is the ER chaperone HSPA5 (GRP78/BiP), which inactivates PERK, IRE1α, and ATF6 under basal conditions but dissociates to engage misfolded proteins during ERS, thereby licensing sensor activation and serving as a master regulator of UPR activation (Pfaffenbach and Lee, 2011; Ibrahim et al., 2019). ERS-induced apoptosis at early lesion sites is a major contributor to SCI pathology (Shi et al., 2018), and ERS markers are rapidly upregulated within hours after injury onset (Ohri et al., 2018).

Accumulating evidence from experimental SCI models confirms robust ERS/UPR activation following injury. In rodent contusion and compression paradigms, canonical markers—including CHOP, ATF4, and spliced XBP1—are markedly upregulated at and surrounding the lesion epicenter within hours and persist for days to weeks (Penas et al., 2007; Ohri et al., 2012). Ultrastructural analyses have revealed dilated ER cisternae and fragmented ER membranes in injured neurons and oligodendrocytes (Ohri et al., 2011). Functionally, persistent PERK–CHOP activation triggers caspase-dependent apoptosis in neurons and oligodendrocytes, contributing to demyelination and neuronal loss (Wang et al., 2013). ERS/UPR pathways also interact with inflammatory cascades; IRE1α can activate NF-κB signaling and facilitate NLRP3 inflammasome assembly, amplifying the pro-inflammatory milieu (Lerner et al., 2012; Shi M. et al., 2021). UPR signaling further regulates autophagy, which may be cytoprotective or cytotoxic depending on stress intensity and duration (Song et al., 2018). Emerging studies suggest that ERS/UPR influences immune cell polarization—particularly M1/M2 phenotype shifts in microglia and infiltrating macrophages—thereby reshaping the post-injury immune microenvironment (Wang et al., 2017; Li et al., 2022). Despite these advances, current knowledge remains confined to individual molecules or isolated signaling branches. A systematic, transcriptome-wide identification of ERS/UPR-related genes and their interaction networks in SCI is lacking, and the relationship between ERS/UPR signaling and immune infiltration patterns remains poorly characterized.

The rapid accumulation of high-throughput transcriptomic data in repositories such as the Gene Expression Omnibus (GEO) offers unprecedented opportunities for systematically dissecting SCI molecular mechanisms. Machine learning approaches, particularly Support Vector Machine–Recursive Feature Elimination (SVM-RFE), can prioritize candidate biomarkers based on their discriminative power. Integrating protein–protein interaction (PPI) network analysis with topological algorithms further enables identification of hub genes occupying central network positions. When coupled with immune infiltration profiling via single-sample Gene Set Enrichment Analysis (ssGSEA), these strategies can reveal links between ERS/UPR signaling and the post-injury immune landscape.

Despite growing recognition of ERS/UPR involvement in SCI, a systematic characterization of ERS/UPR-related differentially expressed genes (DEGs), their hub regulators, and associations with immune infiltration remains absent. To address these gaps, we aimed to: (i) identify ERS- and UPR-related DEGs in SCI using the GEO dataset GSE151371; (ii) apply SVM-RFE to select key feature genes and construct phenotype scoring models; (iii) employ PPI network analysis with five topological algorithms to identify hub genes; (iv) investigate the relationship between ERS/UPR activity and immune cell infiltration via ssGSEA; and (v) validate hub genes using an independent cohort (GSE5296), single-cell RNA-sequencing (scRNA-seq) data (GSE213240), and qRT-PCR in a murine SCI model. Our findings may provide novel insights into ERS/UPR mechanisms in SCI and identify potential biomarkers or therapeutic targets for this devastating condition.

## 2 Materials and methods

### 2.1 Animals

This study used male C57BL/6 J mice (28–32 g) procured from the Laboratory Animal Center of Ningxia Medical University. The rationale for exclusively using male mice in this study was twofold: first, to reflect the higher clinical incidence of SCI in males, and second, to avoid potential experimental variability by mitigating sex-dependent differences in the neuroinflammatory response (Do Rego et al., 2009; Barbiellini Amidei et al., 2022; Zeng et al., 2023). All animals were housed under standard conditions, including a 12-h light/dark cycle, a constant ambient temperature of 21 °C ± 3 °C, and a relative humidity of 50% ± 5%.

### 2.2 Study design

This study employed a multi-phase strategy to systematically identify and validate core genes regulating the ERS and UPR pathways in acute SCI.

Phase I: Hub Gene Discovery. The discovery phase was conducted using the public dataset GSE151371. We first identified DEGs between SCI and sham samples, which were then intersected with MSigDB gene sets to define a pool of ERS/UPR-related candidates. This pool was further refined by SVM-RFE algorithm to select core feature genes. These genes served as the basis for two downstream analyses: 1) the generation of ERS/UPR phenotype scores to correlate with immune cell infiltration quantified by ssGSEA, and 2) the construction of a PPI network to identify central hub genes via topological analysis.

Phase II: Multi-level Validation and Model Construction. This phase validated the identified hub genes, Hspa5 and Dnajc3, through a multi-tiered approach. First, using the independent bulk RNA-seq dataset GSE5296, we confirmed their differential expression and, concurrently, leveraged this cohort to build and evaluate a nomogram diagnostic model via ROC analysis. Second, we examined their expression at single-cell resolution using the scRNA-seq dataset GSE213240. Third, we performed final experimental validation at the transcriptional level by conducting qRT-PCR on spinal cord tissue from our own murine SCI model.

### 2.3 SCI model

An SCI model was established using a standard weight-drop method (Ni et al., 2019). First, mice were anesthetized with isoflurane (2%–4% for induction, 1.5% for maintenance). Following a dorsal midline incision to expose the T10 vertebra, a T10 laminectomy was performed. Subsequently, using an NYU-type impactor, a 10 g metal rod was dropped from a height of 25 mm onto the exposed spinal cord to induce a contusion injury. Care was taken throughout the procedure to prevent any additional compression. After the impact, the muscle and skin were closed in layers. During the surgery and recovery phases, the animals’ body temperature was maintained at 35 °C ± 1 °C with a heating pad.

Sham group animals underwent all surgical procedures, including the T10 laminectomy, but did not receive the spinal cord impact.

### 2.4 RT-qPCR

We employed RT-qPCR to quantify gene transcript abundance. At 24 h post-injury, spinal cord tissues were harvested from the injury epicenter (a 3-mm-long segment) and from segments 3 mm rostral and caudal to the core lesion site. Total RNA was extracted from the collected samples using a TRIzol reagent kit, and its purity and concentration were determined with a Thermo Nanodrop spectrophotometer. After quality confirmation, cDNA was synthesized from the total RNA using a reverse transcription reaction. We then utilized a LightCycler 96 Real-Time PCR System for cDNA amplification and analysis. The relative mRNA expression levels of the target genes were determined according to the comparative Ct (2^−ΔΔCT^) method. Gene expression was normalized to β-actin, which functioned as the endogenous control. A complete list of all primer sequences used is presented in Table 1.

**TABLE 1:** Primers employed in this research.

Gene	Primers	Sequence
Hspa5	Forward primers	GGA​CCA​CCT​ATT​CCT​GCG​TC
	Reverse primers	GGC​TGA​TTA​TCG​GAA​GCC​GT
Dnajc3	Forward primers	TGG​ACT​TTA​CTG​CCG​CAA​GA
	Reverse primers	GCA​GAT​CCT​CTC​CTT​CGA​GC
β-actin	Forward primers	AGC​CAT​GTA​CGT​AGC​CAT​CC
	Reverse primers	GCT​GTG​GTG​GTG​AAG​CTG​TA

### 2.5 Data source

All datasets utilized for the analyses in this study were obtained from publicly accessible databases. Specifically, bulk RNA-sequencing data were downloaded from the GEO database (http://www.ncbi.nlm.nih.gov/geo/), comprising two cohorts: GSE151371 (consisting of 38 acute SCI and 20 sham samples) and GSE5296 (consisting of 27 acute SCI and 24 Sham samples). Additionally, we included a scRNA-seq dataset, GSE213240, which contains 2 moderate SCI and 2 Sham samples.

For the functional enrichment analysis, we retrieved gene sets related to the ERS and the UPR from the MSigDB. Specifically, this included two ERS pathway gene sets (“GOBP_NEGATIVE_REGULATION_OF_RESPONSE_TO_ENDOPLASMIC_RETICULUM_STRESS” and “GOBP_POSITIVE_REGULATION_OF_RESPONSE_TO_ENDOPLASMIC_RETICULUM_STRESS,” totaling 88 genes) and the hallmark UPR gene set (“HALLMARK_UNFOLDED_PROTEIN_RESPONSE,” containing 113 genes).

### 2.6 Screening for ERS- and UPR-associated DEGs

To identify key genes associated with ERS and the UPR in SCI, we first performed a differential expression analysis on the GSE151371 dataset. This analysis was conducted using the limma package in R, and an adjusted *p*-value <0.05 and |logFC| > 0.5 were set as the significance threshold, yielding a list of DEGs.

Next, by intersecting this list of DEGs with the predefined UPR and ERS gene sets, we obtained two highly relevant gene subsets: SCI-UPR DEGs and SCI-ERS DEGs. To further refine the results, both gene subsets were subjected to a rigorous screening using the Wilcoxon test.

Finally, we performed an in-depth functional enrichment study on the final, screened gene list using Gene Ontology (GO) and Kyoto Encyclopedia of Genes and Genomes (KEGG) pathway analysis.

### 2.7 Phenotypic scoring of ERS and UPR and differential enrichment analysis

To select the most representative core genes from the SCI-UPR and SCI-ERS gene sets, we employed the SVM-RFE algorithm for feature selection. Based on these core genes, we then calculated quantitative phenotypic scores for ERS and the UPR for each sample using the GSVA R package with its built-in z-score algorithm.

After obtaining the scores, we promptly assessed the differences between the two scores across samples and evaluated their correlation. Subsequently, both SCI and sham samples were dichotomized into ‘high-score’ and ‘low-score’ groups, using the median of their respective phenotypic scores as the cutoff point.

The final step of the analysis was to perform Gene Set Enrichment Analysis (GSEA) on all DEGs between these high- and low-score groups to investigate their functional differences.

### 2.8 Assessment of immune cell infiltration characteristics

To quantify the level of immune cell infiltration in each sample, we implemented ssGSEA. This computation was performed using the GSVA R package, and the analysis was based on predefined gene sets specific to 28 immune cell types.

After obtaining the immune scores for each sample, we then compared the overall levels, differences, and correlations of immune infiltration between the UPR and ERS groups.

### 2.9 Construction of the PPI network

The set of genes identified by the SVM-RFE algorithm served as the basis for constructing a PPI network. This was achieved by submitting the gene list to the STRING database (v11.5). Subsequent visualization and analysis of the network were performed using Cytoscape software (v3.9.1).

Hub genes were subsequently identified from the network using the Cytoscape plugin cytoHubba. This plugin ranked the genes based on five topological algorithms: BottleNeck, EPC, Closeness, Betweenness, and ClusteringCoefficient. An intersection analysis of the top 10 genes from each algorithm was performed, which yielded two final hub genes.

### 2.10 Establishment and performance evaluation of a diagnostic model

To establish the predictive role of *Hspa5* and *Dnajc3*, we first examined their expression performance in the external cohort GSE5296. Based on the expression data of these two genes, we performed a logistic regression analysis and developed a nomogram prediction model using the rms R package.

To ultimately assess the diagnostic accuracy of this gene signature, we generated a ROC curve using the pROC R package and used it to evaluate the model’s diagnostic efficiency.

### 2.11 scRNA-seq data analysis

To validate the expression of our hub genes at the single-cell level, we utilized the scRNA-seq dataset GSE213240 (2 SCI vs. 2 Sham samples). Data analysis was primarily conducted using the Seurat R package (v4.4.0). First, we performed quality control on the raw count matrix. Subsequently, the data was normalized using the log-normalization (“LogNormalize”) method, and batch effects were corrected using a canonical correlation analysis integration algorithm. To assess the expression of the hub genes *Dnajc3* and *Hspa5*, we generated violin plots to compare their overall expression levels between the SCI and sham groups.

### 2.12 Statistical analysis

Statistical analyses were conducted using R software (v4.3.3), while GraphPad Prism (version 9.3) was employed specifically for the analysis of experimental data. Bioinformatic analyses involved using the Wilcoxon test to evaluate differences between two groups of continuous variables, while associations among variables were quantified via Spearman’s correlation. For qRT-PCR data, results are presented as the mean ± standard deviation (SD), and statistical differences between groups were assessed using Student’s t-test. Statistical significance across all analyses was defined by a *p*-value of less than 0.05. Graphical representations of the data were produced utilizing the ggplot2 package (R) or GraphPad Prism software.

## 3 Results

### 3.1 Defining the ERS/UPR-linked DEGs following SCI

**FIGURE 1:**
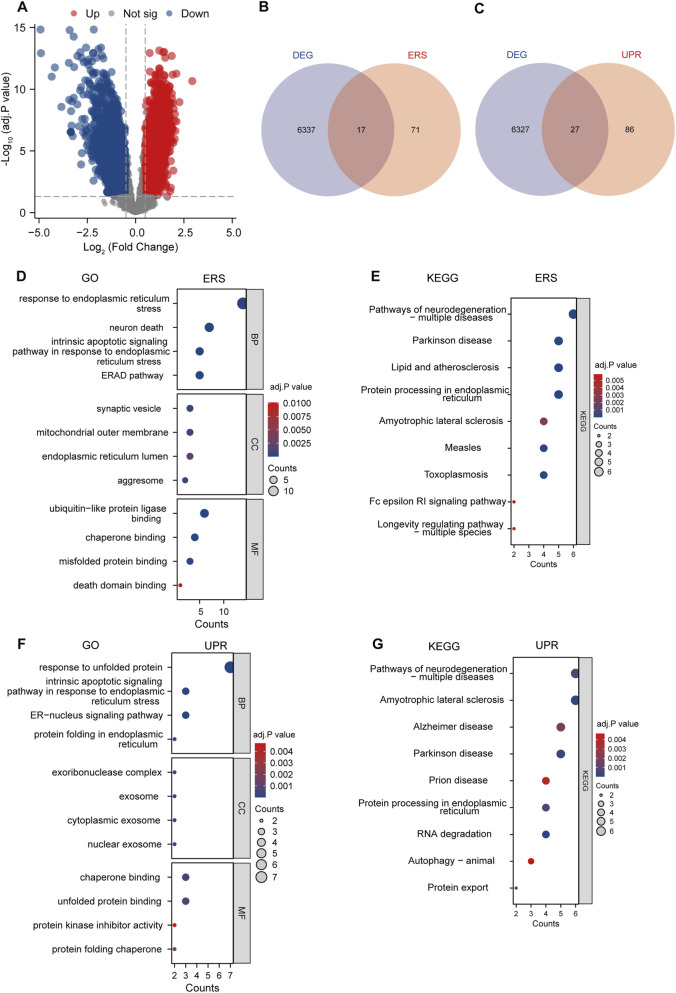
Discovery and Functional Annotation of ERS/UPR-Related DEGs in SCI. **(A)** Volcano plot showing DEGs in SCI samples. **(B,C)** Venn diagrams showing the intersection of DEGs with ERS and UPR gene sets. **(D,E)** Functional annotation and pathway mapping for ERS-related DEGs in SCI. **(F,G)** Functional annotation and pathway mapping for UPR -related DEGs in SCI.

A comparison of the transcriptomes from the SCI and sham groups revealed 6,354 DEGs, comprising 3,399 upregulated and 2,955 downregulated genes (Figure 1A). To define their relevance to ERS and the UPR, we intersected our list of DEGs with established gene sets, which yielded 17 ERS-related and 27 UPR-related DEGs (Figures 1B,C).

The differential expression of candidate genes between the SCI and sham groups was subsequently validated using a Wilcoxon test. We excluded three genes from the final dataset—namely, *FCGR2B*, *APP*, and *LRRK2*—as their expression levels lacked significant differential changes (adjusted *p* > 0.05). This filtering step resulted in a final list of 41 high-confidence DEGs for subsequent analysis (Supplementary Figure S1A,B).

These 41 DEGs were then subjected to GO and KEGG pathway enrichment analyses. For the SCI-ERS DEGs, significantly enriched GO biological processes included “response to endoplasmic reticulum stress,” “neuron death,” and “intrinsic apoptotic signaling pathway in response to endoplasmic reticulum stress” (Figure 1D). KEGG pathway enrichment analysis revealed that the most significantly enriched pathways were “Pathways of neurodegeneration - multiple diseases,” “Parkinson disease,” and “Lipid and atherosclerosis” (Figure 1E).

For the SCI-UPR DEGs, GO analysis revealed significant enrichment in functions such as “response to unfolded protein” and “intrinsic apoptotic signaling pathway in response to endoplasmic reticulum stress” (Figure 1F). Correspondingly, KEGG pathway analysis showed primary enrichment in “Pathways of neurodegeneration - multiple diseases,” and “Alzheimer disease” (Figure 1G).

### 3.2 Functional overlap analysis of ERS and UPR phenotype scores

**FIGURE 2:**
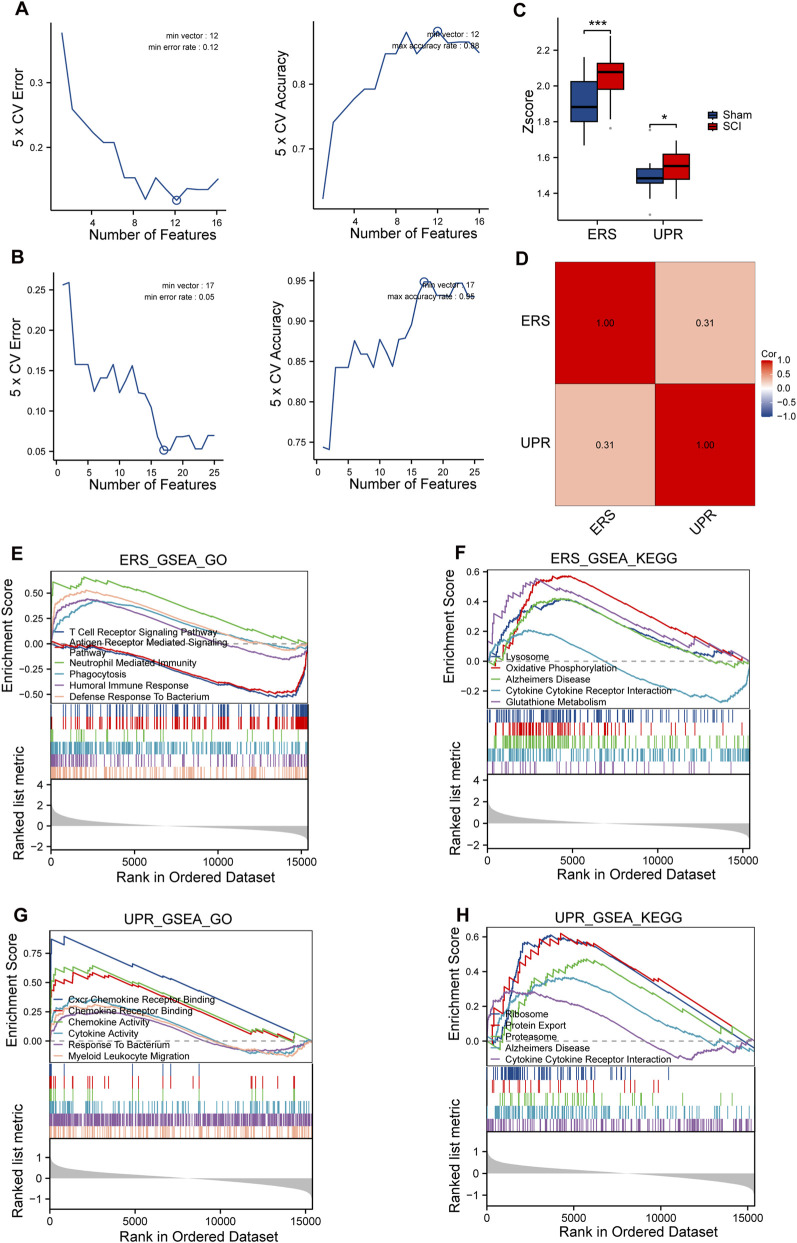
Development and Functional Validation of ERS and UPR Phenotype Scores in SCI. **(A)** Identification of key ERS-related genes via the SVM-RFE algorithm. **(B)** Identification of key UPR-related genes via the SVM-RFE algorithm. **(C)** Comparison of ERS and UPR phenotype scores between SCI and Sham groups. **(D)** Analysis of the relationship between ERS and UPR phenotype scores. **(E,F)** Functional characterization of the ERS gene signature. **(G,H)** Functional characterization of the UPR gene signature.

The SVM-RFE algorithm was utilized to select a panel of key genes with the highest predictive accuracy for SCI. From the 17 SCI-ERS DEGs, 12 key genes were selected: *HSPA1A*, *ALOX5*, *BCAP31*, *CLU*, *USP13*, *HSPA5*, *ATF6*, *GRINA*, *SIK2*, *LPCAT3*, *AQP11*, and *APP* (Figure 2A). Similarly, from the 27 SCI-UPR DEGs, 17 key genes were identified: *EXOSC4*, *SEC11A*, *TSPYL2*, *EXOSC2*, *ATF6*, *WIPI1*, *PDIA5*, *HSPA5*, *KIF5B*, *TUBB2A*, *DDX10*, *NOLC1*, *DDIT4*, *CEBPB*, *FKBP14*, *IFIT1*, and *DNAJC3* (Figure 2B).

Using these selected genes, we constructed phenotype scoring models based on the z-score algorithm. The results indicated that SCI samples exhibited significantly elevated phenotype scores for both ERS and the UPR (Figure 2C). Furthermore, a strong positive correlation was observed between these two phenotype scores (r = 0.31) (Figure 2D).

To further explore their functional implications, we performed differential analysis and Gene Set Enrichment Analysis (GSEA). The results revealed that in the high-ERS score group, pathway activities for the lysosome and oxidative phosphorylation were significantly increased. Similarly, elevated activities of ribosome and proteasome were also observed in the high-UPR score group. These findings further underscore the close association among ERS, the UPR, and immune-metabolic functions in the context of SCI (Figures 2E–H).

### 3.3 Correlation analysis of immune infiltration with ERS and UPR

**FIGURE 3:**
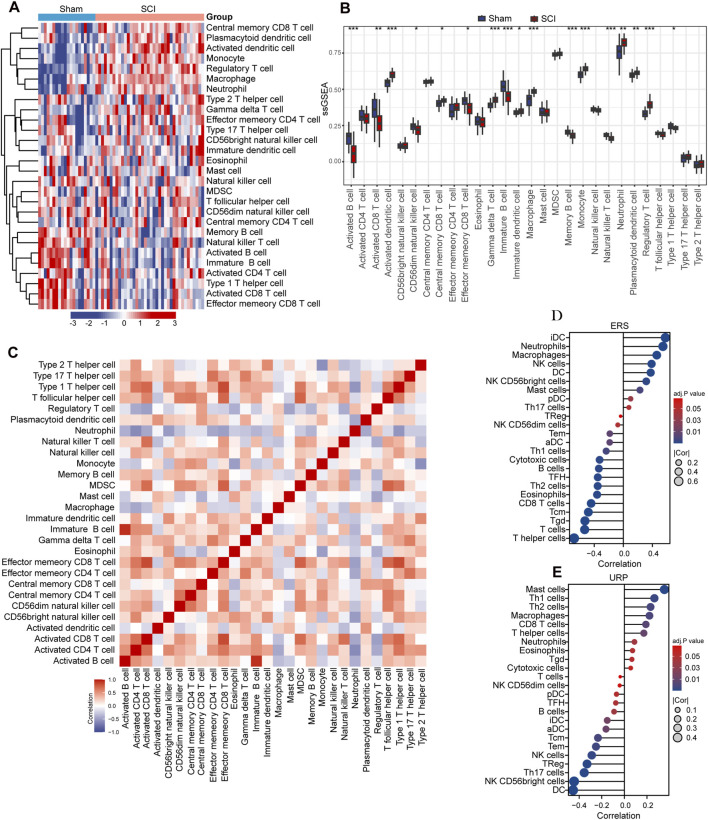
Analysis of immune infiltration based on the ssGSEA algorithm. **(A)** A heatmap displaying the abundance of 28 immune cell types. **(B)** Comparison of immune cell enrichment scores between the SCI and Sham groups. **(C)** Analysis of the relationships among different immune cell populations. **(D,E)** A correlation plot illustrating the association between phenotype scores and immune cell abundance.

To better understand the contribution of immune cells to SCI pathogenesis, we characterized the landscape of immune infiltration (Figure 3A). Of the 28 distinct immune cell types profiled, 17 were significantly altered in abundance between the SCI and Sham groups. Specifically, the infiltration levels of Activated CD8 T cells, CD56dim natural killer cells, Effector memory CD8 T cells, Immature B cells, Memory B cells, Natural killer T cells, and Type 1 T helper cells were downregulated in the SCI group. Conversely, the levels of Activated dendritic cells, Central memory CD8 T cells, Gamma delta T cells, Immature dendritic cells, Macrophages, Monocytes, Neutrophils, Plasmacytoid dendritic cells, and Regulatory T cells were upregulated (Figure 3B). The abundance of these diverse immune cell subsets was found to be highly inter-correlated (Figure 3C).

The interplay between the immune infiltration landscape and the signaling activity of the ERS and UPR pathways was also examined. The results indicated that the ERS score was positively correlated with immature dendritic cells (iDCs), neutrophils, and macrophages, while showing a negative correlation with T helper cells, T cells, and CD8 T cells. The UPR score was positively correlated with mast cells, Th1 cells, and macrophages, but was negatively correlated with dendritic cells (DCs), NK CD56bright cells, and Th17 cells (Figures 3D,E).

### 3.4 Identification of hub genes and construction of a diagnostic model for SCI

**FIGURE 4:**
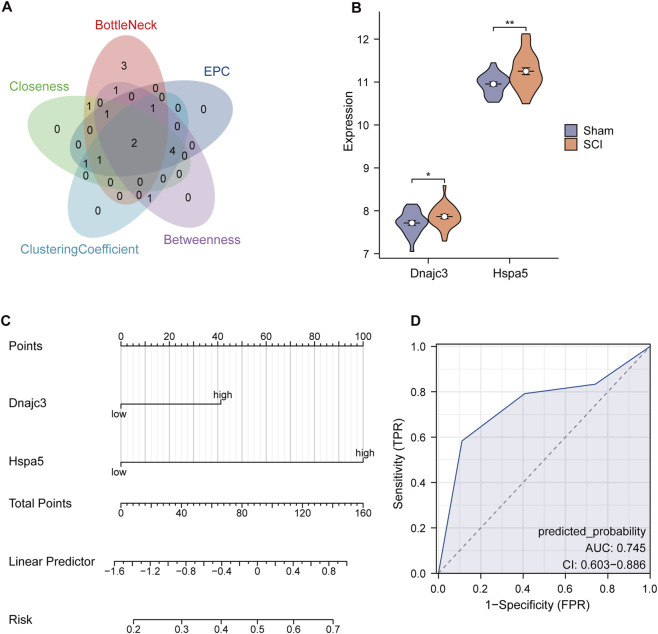
Identification, validation, and diagnostic model construction of hub genes. **(A)** A Venn diagram showing the intersection of candidate genes identified through five different topological algorithms (BottleNeck, EPC, Closeness, Betweenness, and Clustering Coefficient), which pinpointed *Dnajc3* and *Hspa5* as the two hub genes. **(B)** In the external validation cohort GSE5296, the expression levels of both *Dnajc3* and *Hspa5* were significantly higher in the SCI group compared to the sham group. **(C)** A nomogram model constructed based on the expression levels of *Dnajc3* and *Hspa5* to predict the probability of SCI. **(D)** ROC curve analysis of the diagnostic model, which yielded an AUC of 0.745.

To identify the most critical genes from the 29 candidates previously screened by the SVM-RFE algorithm, we performed a PPI network analysis. The PPI network was first constructed using the STRING database and subsequently imported into Cytoscape for topological analysis. Five distinct algorithms—BottleNeck, EPC, Closeness, Betweenness, and Clustering Coefficient—were employed to select the top 10 ranked genes from the network, respectively (Supplementary Table S1). To pinpoint the most robust and central genes, we performed an intersection analysis of the gene sets from all five algorithms. This rigorous screening process ultimately identified two highly central hub genes: Dnajc3 and Hspa5 (Figure 4A).

To test the robustness of this finding, we validated the expression levels of Dnajc3 and Hspa5 in an independent, external dataset (GSE5296). Consistent with our primary analysis, the analysis revealed that both genes were significantly upregulated in SCI samples compared to the sham group in this validation cohort, further strengthening their close association with the acute pathological processes of SCI (Figure 4B).

Given the central roles of Dnajc3 and Hspa5 and their confirmed upregulation post-SCI, we proceeded to construct a diagnostic model to evaluate their potential as a combined biomarker signature. A nomogram was established that integrates the expression levels of Dnajc3 and Hspa5 to provide a quantitative probability of a sample being classified as an SCI case. As depicted in the nomogram, higher expression levels of both genes correspond to higher point values; the summation of these points yields a total score that correlates with a higher diagnostic probability (Figure 4C). This user-friendly graphical model provides an intuitive tool for assessing the SCI-associated molecular signature based on these two key genes.

Subsequently, the diagnostic performance of the Dnajc3-Hspa5 nomogram was evaluated using a Receiver Operating Characteristic (ROC) curve. The model demonstrated strong discriminative power, achieving an AUC of 0.745. This result indicates a high degree of accuracy in distinguishing SCI samples from control samples, thereby validating the diagnostic utility of this two-gene signature (Figure 4D).

### 3.5 Validation of hub gene expression at the single-cell and transcriptional levels

**FIGURE 5:**
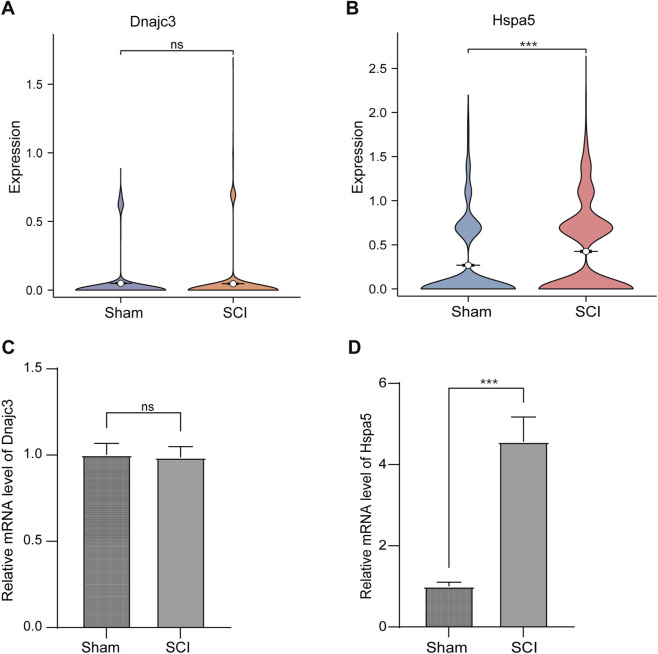
Validation of hub gene expression at the single-cell and transcriptional levels. **(A,B)** scRNA-seq analysis revealed that *Dnajc3* expression was not significantly different, whereas *Hspa5* expression was significantly upregulated in the SCI group compared to the sham group. **(C,D)** Validation by qRT-PCR demonstrated no significant difference in the mRNA levels of *Dnajc3* between groups (n = 5), but confirmed that *Hspa5* mRNA levels were significantly elevated in the SCI group (n = 5). ns, not significant; ***p < 0.001.

To further investigate the specific roles of our identified hub genes, *Dnajc3* and *Hspa5*, and to validate our bioinformatics findings at a higher resolution, we analyzed their expression using a scRNA-seq dataset of spinal cord tissue. In this granular, cell-level analysis, we observed a significant upregulation of *Hspa5* expression in cells from the SCI group compared to the sham group (Figure 5B). Interestingly, single-cell analysis revealed no significant difference in the expression of *Dnajc3* between the two comparison groups (Figure 5A). This finding suggests that while *Dnajc3* may play a role within the PPI network, its transcriptional dysregulation in SCI might not be as widespread or significant at the single-cell level compared to *Hspa5*, or it may exhibit cell-type specific expression not broadly captured.

To confirm these findings at the bulk tissue transcriptional level, we performed qRT-PCR on spinal cord tissue samples from our own SCI and sham experimental animal models. The results of the qRT-PCR analysis precisely mirrored our single-cell data: *Hspa5* mRNA levels were robustly and significantly elevated in the SCI group relative to the sham controls (Figure 5C), whereas *Dnajc3* mRNA levels remained unchanged (Figure 5D). This consistent lack of significant upregulation for *Dnajc3* across high-resolution and experimental validation further challenges its reliability as a direct transcriptional biomarker for SCI.

Taken together, these complementary validation experiments strongly indicate that while both *Dn*ajc3 and *Hspa5* were identified as central nodes in the protein-protein interaction network, Hspa5 emerges as the more dominant and consistent biomarker for the unfolded protein response in the *in vivo* environment following SCI. The consistent upregulation of *Hspa5* across bulk RNA-seq, single-cell sequencing, and qRT-PCR analyses highlights its pivotal role in mediating endoplasmic reticulum stress after spinal cord injury.

## 4 Discussion

In this study, we systematically characterized ERS and UPR–related transcriptional changes after acute SCI, quantified ERS/UPR activity at the sample level, linked these phenotypes to immune-metabolic remodeling, and identified *Hs*pa5 as a robust and dominant hub gene, consistently and significantly upregulated across multiple validation platforms, through multi-level validation.

A major implication of our work is that ERS/UPR signaling represents a focused yet critical component of the post-SCI transcriptomic response rather than a diffuse by-product of global injury. GO enrichment specifically highlighted “neuron death” and the “intrinsic apoptotic signaling pathway in response to ER stress”, in agreement with prior mechanistic studies demonstrating that ERS-induced apoptosis is a key driver of secondary injury and contributes to neuronal and glial loss after SCI (Soni et al., 2023). KEGG analysis further revealed that ERS/UPR-related DEGs were significantly enriched in “Pathways of neurodegeneration–multiple diseases”, including Alzheimer’s disease, and Parkinson’s disease. These data support the concept that acute neurotrauma and chronic neurodegenerative disorders, despite distinct etiologies and temporal profiles, converge on shared molecular machinery of proteostasis imbalance and maladaptive cellular stress (Hetz and Saxena, 2017). Our transcriptomic findings also provide high-throughput reinforcement of earlier work focused on individual ERS/UPR components in SCI models, where upregulation of canonical markers such as Ddit3 (CHOP) and spliced Xbp1 (XBP1s) has been repeatedly demonstrated at both mRNA and protein levels in the injured spinal cord (Gao et al., 2023; Xiao et al., 2026). Our study corroborates the prevalence and significance of these molecular events at a broader, gene-set level.

A second key advance lies in the quantitative assessment of ERS and UPR activity using phenotype scores derived from SVM-RFE–selected genes. Both ERS and UPR scores were significantly increased in SCI samples and showed a positive correlation, indicating that they capture tightly coupled, synergistic dimensions of the post-injury stress response. GSEA based on stratified scores revealed that high-ERS samples were enriched for “lysosome” and “oxidative phosphorylation”, pointing to ER–mitochondria crosstalk and activation of autophagy-lysosomal pathways that promote damaged organelle and protein clearance (Hetz and Papa, 2018; Liu et al., 2022). In parallel, high-UPR samples showed enhanced “ribosome” and “proteasome” activity, reflecting the classical UPR strategy of transiently restraining protein synthesis while accelerating ER-associated degradation (ERAD) to remove misfolded proteins (Brodsky, 2012). Together, these findings support a model in which intense ER stress imposes a UPR-mediated reprogramming of protein synthesis, degradation, and energy metabolism, thereby steering the balance between adaptive survival and apoptotic cell death in the injured spinal cord (Walter and Ron, 2011).

Beyond intracellular proteostasis, our data indicate that ERS/UPR activation is intimately linked to immune microenvironment remodeling in SCI. ssGSEA across 28 immune cell subsets showed that 17 populations were significantly altered in SCI compared with Sham: monocytes, macrophages, neutrophils, several dendritic cell subsets, and regulatory T cells were increased, whereas activated CD8 T cells, CD56dim NK cells, effector memory CD8 T cells, Th1 cells, and B-cell subsets were decreased. This pattern mirrors the transition from an adaptive and cytotoxic effector–dominated landscape toward one characterized by innate myeloid cells and immunoregulatory phenotypes that has been described in acute SCI (Beck et al., 2010). Correlation analysis further revealed that the ERS score was positively associated with immature dendritic cells, neutrophils, and macrophages but negatively associated with T helper and CD8 T cells, whereas the UPR score was positively associated with mast cells, Th1 cells, and macrophages but negatively associated with dendritic cells, CD56bright NK cells, and Th17 cells. Mechanistically, these associations are consistent with experimental evidence that ERS/UPR signaling can trigger pro-inflammatory cytokine production through the IRE1α–NF-κB–NLRP3 inflammasome axis, thereby promoting recruitment and activation of neutrophils and macrophages (Kaneko et al., 2003; Menu et al., 2012), and that UPR pathways modulate microglial/macrophage M1/M2 polarization following CNS injury (Valenzuela et al., 2012). Conversely, the negative correlations between ERS scores and T-cell subsets likely reflect suppression or exhaustion of adaptive effector cells within a high-stress, myeloid-dominant milieu in acute SCI (Cubillos-Ruiz et al., 2015). Collectively, these findings support an “ERS/UPR–immune axis” framework in which ERS/UPR acts as a central signaling hub that not only integrates proteostatic stress but also actively sculpts the immune landscape after SCI.

Within the ERS/UPR-related gene network, we identified *Dnajc3* and *Hspa5* as central hub genes using a PPI-based topological approach that combined five complementary algorithms. Both genes were significantly upregulated in the independent external dataset GSE5296, supporting their robustness as injury-associated markers. However, multi-level validation revealed a clear divergence between them. *Hspa5*, encoding GRP78/BiP, showed consistent and significant upregulation across bulk RNA-seq, scRNA-seq, and qRT-PCR in our animal model, firmly establishing it as a dominant marker of UPR activation in acute SCI. GRP78/BiP is the canonical master regulator of the UPR: under homeostatic conditions, it binds to and represses the three UPR sensors IRE1α, PERK, and ATF6, and upon accumulation of misfolded proteins, it dissociates to initiate downstream UPR cascades (Cubillos-Ruiz et al., 2015). Notably, *Hspa5* upregulation has also been frequently reported in Alzheimer’s disease and Parkinson’s disease (Hetz and Saxena, 2017; Pazi et al., 2024), which is concordant with our enrichment of ERS/UPR DEGs in “Pathways of neurodegeneration–multiple diseases” and further supports *Hspa5* as a shared molecular node bridging acute neurotrauma and chronic neurodegeneration. In contrast, although *Dnajc3* emerged as a PPI hub and was upregulated in bulk datasets, it did not show significant changes in either scRNA-seq or qRT-PCR. This discrepancy is biologically plausible: network centrality reflects topological importance in the interactome, not necessarily large fold-changes at the transcript level (He and Zhang, 2006). As an HSP40 co-chaperone, Dnajc3 augments the ATPase activity of GRP78 and facilitates protein folding in the ER (Kampinga and Craig, 2010). Its strong functional connectivity can confer hub status, while its transcriptional regulation may be cell-type-specific, time-restricted, or under post-transcriptional control, making it less detectable by bulk and endpoint assays at the sampled time points. Therefore, while *Dnajc3’s* topological importance is evident, its direct utility as a robust biomarker and its potential as a therapeutic target in the ERS/UPR-immune axis warrant further comprehensive functional studies.

Based on the central roles of *Dnajc3* and *Hspa5*, we developed a logistic-regression–based nomogram that integrates the expression of these two genes and achieved an AUC of 0.745 for distinguishing SCI from Sham samples. This performance indicates moderate-to-good discrimination and provides a preliminary, visually interpretable tool that links molecular readouts to SCI probability. From a translational perspective, current SCI diagnosis and severity grading rely largely on imaging and neurological assessments such as the ASIA scale, and there is a conspicuous lack of objective, molecular-level biomarkers (Marshall, 2017). The *Dnajc3*–*Hspa5* two-gene signature proposed here thus offers candidate targets for the future development of ERS/UPR-related biomarkers, particularly in clinically accessible specimens such as peripheral blood or cerebrospinal fluid (CSF). Supporting this possibility, GRP78/BiP has been detected as a secreted protein in serum and CSF under various pathological conditions (Ni et al., 2011), suggesting that *Hspa5*-derived products could be explored as liquid biopsy markers for SCI. These findings further suggest that directly targeting *Hspa5* or modulating the ERS/UPR-immune axis could offer novel therapeutic strategies for spinal cord injury. Future preclinical studies could explore the *in vivo* effects of inhibiting or activating *Hspa5*, or intervening pharmacologically to regulate the activity of the ERS/UPR pathway, to assess their potential in improving SCI outcomes. At the same time, an AUC of 0.745 is below the threshold generally expected for standalone clinical decision-making, and it is likely that incorporation of additional molecular markers or clinical variables will be necessary to achieve the high discrimination (e.g., AUC >0.9) required for routine use.

This study has several strengths. Methodologically, we integrated a large-sample bulk RNA-seq dataset (GSE151371), an independent external cohort (GSE5296), a scRNA-seq dataset (GSE213240), and *in vivo* qRT-PCR validation, thereby constructing a multi-layered evidence framework that enhances the robustness of our conclusions. Analytically, we combined classical differential expression with SVM-RFE feature selection, PPI network topology, and ssGSEA immune infiltration profiling, providing more systematic insights at both the feature and network levels than conventional DEG-only approaches (Guyon et al., 2002). Conceptually, by directly linking ERS/UPR activation to immune microenvironment remodeling, our work introduces a relatively novel perspective into SCI bioinformatics research. Nonetheless, important limitations should be acknowledged. Firstly, the experimental validation in this study involved a limited number of animal samples, which might impact the generalizability of the results. Secondly, our findings are primarily correlative at the transcriptomic level, and we did not perform functional experiments (e.g., targeted knockdown or overexpression of *Hspa5*, pharmacological modulation of UPR branches) to dissect causal roles in SCI pathology. Thirdly, although we confirmed *Hspa5* upregulation in spinal cord tissue, we did not assess its performance as a biomarker in peripheral blood or CSF, which will be essential for clinical translation. Fourthly, our animal experiments exclusively used male mice, which, despite controlling for hormone-related variability, limits the direct generalizability of our findings to females due to known sex differences in SCI pathophysiology. Future studies should therefore incorporate temporally resolved and cell-type–specific functional experiments, and systematically evaluate *Hspa5* and related ERS/UPR components as liquid biopsy candidates, to bridge the gap between mechanistic insights and practical diagnostic or therapeutic applications.

## 5 Conclusion

In conclusion, this study systematically characterized ERS/UPR-related transcriptional responses in SCI and revealed their intimate association with immune cell infiltration and immuno-metabolic pathways. *Hspa5*, as a consistently upregulated hub gene validated across multiple dimensions, represents a promising biomarker and potential therapeutic target on the ERS/UPR axis. These findings provide a valuable theoretical framework and candidate molecular basis for the future development of ERS/UPR-targeted intervention strategies to improve outcomes in SCI.

## Data Availability

Publicly available datasets were analyzed in this study. This data can be found here: The datasets (GSE151371, GSE5296, and GSE213240) used in this study are available in the GEO database (https://www.ncbi.nlm.nih.gov/geo/).
