# Supplementary material for: Integrative analysis identifies Hspa5 as a key regulator of the ERS/UPR-immune axis in spinal cord injury
Source: Front Genet. 2026 Jun 19;17:1833119. doi: 10.3389/fgene.2026.1833119 (PMC13327658; doi:10.3389/fgene.2026.1833119)
Supplement: Supplementary file 2 [file DataSheet1.docx]

Supplementary Material


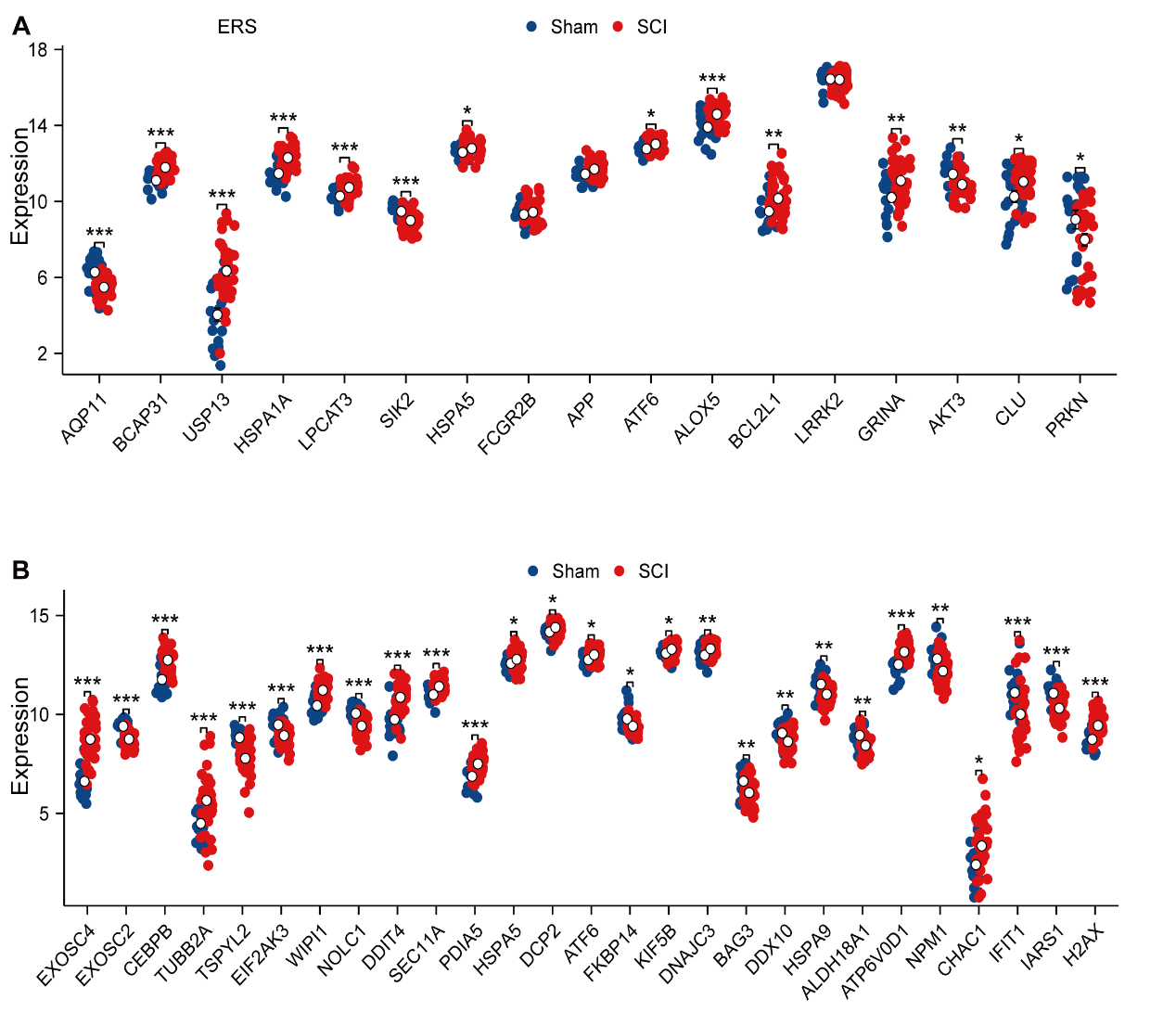


**Supplementary Figure 1.** Expression of identified SCI-ERS DEGs (A) and SCI-UPR DEGs (B). **p* < 0.05, ***p* < 0.01 and ****p* < 0.001.
